# Treatment-resistant depression: molecular mechanisms and management

**DOI:** 10.1186/s43556-024-00205-y

**Published:** 2024-10-17

**Authors:** Mayanja M. Kajumba, Angelina Kakooza-Mwesige, Noeline Nakasujja, Deborah Koltai, Turhan Canli

**Affiliations:** 1 Department of Mental Health and Community Psychology, Makerere University, P. O. Box 7062, Kampala, Uganda; 2 Department of Pediatrics and Child Health, Makerere University College of Health Sciences, Kampala, Uganda; 3 Department of Pediatrics and Child Health, Mulago National Referral Hospital, Kampala, Uganda; 4 Department of Psychiatry, School of Medicine, Makerere University College of Health Sciences, Kampala, Uganda; 5 Duke Division of Global Neurosurgery and Neurology, Department of Neurosurgery, Durham, NC USA; 6 Department of Neurology, Duke University School of Medicine, Durham, NC USA; 7 Department of Psychiatry and Behavioral Sciences, Duke University School of Medicine, Durham, USA; 8 Department of Psychology, Stony Brook University, New York, USA; 9 Department of Psychiatry, Stony Brook University, New York, USA

**Keywords:** TRD, Molecular, Mechanisms, Pharmacological, Non-pharmacological, Management

## Abstract

Due to the heterogeneous nature of depression, the underlying etiological mechanisms greatly differ among individuals, and there are no known subtype-specific biomarkers to serve as precise targets for therapeutic efficacy. The extensive research efforts over the past decades have not yielded much success, and the currently used first-line conventional antidepressants are still ineffective for close to 66% of patients. Most clinicians use trial-and-error treatment approaches, which seem beneficial to only a fraction of patients, with some eventually developing treatment resistance. Here, we review evidence from both preclinical and clinical studies on the pathogenesis of depression and antidepressant treatment response. We also discuss the efficacy of the currently used pharmacological and non-pharmacological approaches, as well as the novel emerging therapies. The review reveals that the underlying mechanisms in the pathogenesis of depression and antidepressant response, are not specific, but rather involve an interplay between various neurotransmitter systems, inflammatory mediators, stress, HPA axis dysregulation, genetics, and other psycho-neurophysiological factors. None of the current depression hypotheses sufficiently accounts for the interactional mechanisms involved in both its etiology and treatment response, which could partly explain the limited success in discovering efficacious antidepressant treatment. Effective management of treatment-resistant depression (TRD) requires targeting several interactional mechanisms, using subtype-specific and/or personalized therapeutic modalities, which could, for example, include multi-target pharmacotherapies in augmentation with psychotherapy and/or other non-pharmacological approaches. Future research guided by interaction mechanisms hypotheses could provide more insights into potential etiologies of TRD, precision biomarker targets, and efficacious therapeutic modalities.

## Introduction

Depression is one of the most common among mental disorders, affecting more than 300 million people worldwide, and can be profoundly disabling [1, 2]. It is among the leading causes of morbidity and mortality [3], and the past 60 years have been devoted to extensive research, investigating its etiological mechanisms and evidence-based treatment strategies [4, 5]. This has led to the discovery of various antidepressant therapies including serotonin reuptake inhibitors (SSRIs), serotonin and norepinephrine reuptake inhibitors (SNRIs), tricyclic antidepressants (TCA), monoamine oxidase inhibitors (MAOIs), atypical antidepressants [6], adjunctive agents and non-pharmacological strategies [7]. However, despite the extensive research efforts and discoveries, antidepressants are still not effective for more than 60% of the patients [8, 9], and a trial-and-error treatment approach is currently used for most patients [10]. The treatment of depression typically involves antidepressant dose optimization, switching, combination, and/or augmentation with other agents, such as atypical antipsychotics [11–14], ketamine [15, 16], lithium [7, 17], Chinese herbal medicine [18, 19], and/or non-pharmacological therapies, like neurostimulation [16, 20], and psychotherapy [21]. But, whether used individually or in combination, none of these therapies seems effective for most depressed patients, and the molecular targets for efficacious antidepressant therapy are still unknown.

The prevailing assumption over the past decades has been that depression is a result of the depletion of monoamines, and most of the conventional antidepressants primarily target serotonin (5-hydroxytryptamine, 5-HT), norepinephrine (NE) [22, 23] and to a lesser extent, dopamine (DA) [24]. Interestingly however, monoaminergic neurotransmitter systems account for only less than 10% of the central nervous system activity [25] and monoamine-based antidepressants are not effective for the majority of depressed patients [26], and when effective, they have a delayed onset of therapeutic action, and require chronic administration [27]. The therapeutic effects of antidepressants are not only realized after several weeks or months [28], but the response also varies from individual to individual [28]. More than 50% of depressed individuals do not respond to conventional first-line treatment [1, 29–31] and about 30% remain unresponsive to several trials of different antidepressant medications [32]. Furthermore, the patients who respond to treatment have a 60% chance of relapse following their first episode [33], and as many as 50% [34] remain burdened with residual symptoms [12, 35]. These residual symptoms tend to be mainly somatic and include reduced appetite, psychomotor retardation [36], fatigue, muscle ache, insomnia, reduced sexual desire, and inability to experience pleasure (anhedonia), as well as ongoing low mood, anxiety, guilt, loss of interest, and irritability [37]. Thus, even among patients who achieve remission, there may be some residual symptoms, which not only impair the patients’ functioning but also potentially contribute to the recurrence and chronicity of depression.

Treatment-resistant depression (TRD) refers to a subtype of depression for which the patients remain unresponsiveness to at least two adequately administered conventional antidepressant treatments [32, 38, 39], with optimal adherence and treatment duration [31, 40]. There is no agreement on the exact definition of an adequate dosage and duration, but most experts consider a TRD diagnosis, if patients experience a less than 50% reduction in depressive symptoms after 4 weeks [32, 41, 42] of optimal use of at least two prescribed antidepressant treatments [31, 40, 41]. However, a number of researchers and clinicians indicate that a TRD diagnosis should be made if a patient is unresponsiveness for a minimal duration of 6 – 8 weeks on pharmacotherapy, and 10 –12 weeks on trial psychotherapy [32, 43]. Overall, a less than 50% symptom reduction is a consistently used criterion in assessing antidepressant treatment resistance, and TRD patients are categorized as either partial responders (25–50% improvement) or non-responders (< 25% improvement) [44]. Currently, there are no known subtype-specific biomarkers to help in the determination of the appropriate treatment for TRD [10], which is a challenge to clinicians whose patients remain with depressive symptoms even after several antidepressant treatment trials.

The consistent finding that antidepressants are not effective for the majority of patients, has led some researchers to the conclusion that these drugs have minimal value and that they should not be prescribed for depressed patients, except where the potential benefits outweigh the adverse effects [2]. Depression is, however, a heterogeneous disorder [45–47] whose proximal causes may lie anywhere on the spectrum of social, psychological, or biological variables for any given patient, with significant individual differences in the underlying mechanisms, and required treatment. This review reveals different pathogenic mechanisms and potential therapeutic targets, as well as the currently used pharmacological and non-pharmacological strategies in managing TRD. It also presents evidence on the efficacy of the recently approved and emerging TRD therapies and insights about future directions.

## Molecular mechanisms of treatment-resistant depression

### Monoamine neurotransmitter interactions in antidepressant treatment resistance

**Fig. 1:**
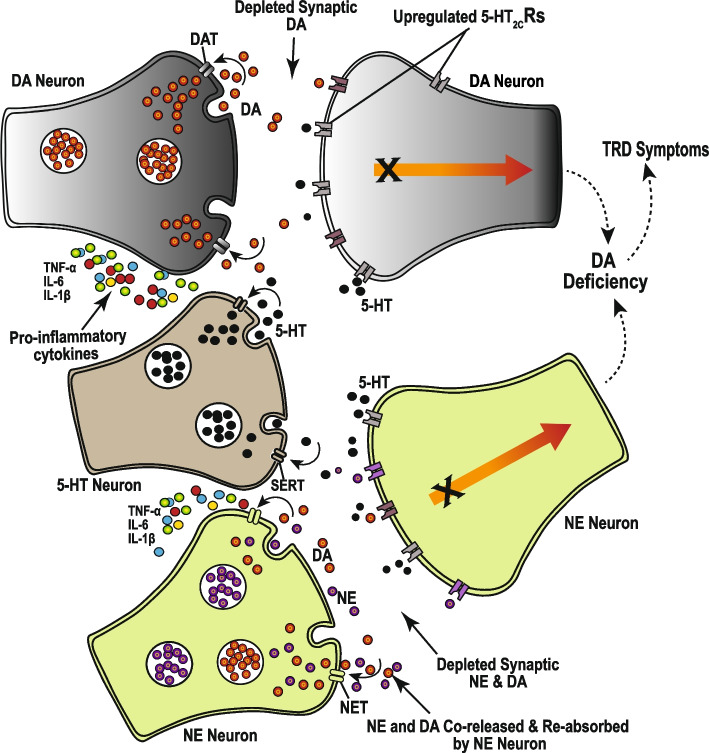
Monoamine neurotransmitter interactions in depression and treatment response. The underlying mechanisms for depression, involve a complex interaction among the monoaminergic neurotransmitter systems, and neuroinflammation. Pro-inflammatory cytokines such as TNF-α, IL-6, and IL-1β increase the sensitivity of 5-HT, NE, and DA reuptake transporters, which results in increased reabsorption and depletion of synaptic 5-HT, NE, and DA. The depletion of 5-HT results in an upregulation of inhibitory 5-HT_2C_ receptors, which during situations of increased 5-HT release contributes to enhanced tonic inhibition of DA and NE neurons, and worsened depression. This is observed in SSRI-induced increase in 5-HT levels, which results in a DA deficiency, characterized by motivational deficits, psychomotor retardation, anhedonia, decreased emotional responsivity, and other depression symptoms. These symptoms are induced by, and thus resistant to SSRIs, but could be alleviated through augmentation of SSRIs with DA neurotransmission enhancing agents, or any mechanisms that result in the desensitization of 5-HT_2C_Rs. The desensitization of 5-HT_2C_Rs in response to prolonged exposure to high 5-HT levels, could be required for therapeutic action and may account for delayed onset of the therapeutic effects of SSRIs and SNRIs

Contrary to the monoamine hypothesis, depression seems not to just be a consequence of neurotransmitter deficiency, but rather due to a complex interaction among monoamine neurotransmitter systems. Indeed, depending on the nature of serotonin (5-HT) receptors involved in the interaction with other monoamines, depression may be a result of either a decrease or an increase in 5-HT levels. For example, both the noradrenergic and dopaminergic neurons [48] in the prefrontal cortex (PFC) and other regions involved in depression are under tonic inhibition by 5-HT, which modulates dopamine (DA) activity, in all three major dopaminergic pathways [49]. Several 5-HT receptors are involved in the 5-HT-DA interaction, including 5-HT_1A_R, 5-HT_1B_R, 5-HT_2A_R, 5-HT_3_R, and 5-HT_4_R, whose agonists enhance DA release, and the 5-HT_2C_R, whose agonists increase tonic inhibit, and decrease DA release [50, 51]. Furthermore, DA is a precursor for norepinephrine (NE), and it is co-released with NE by NE neurons [52] (Fig. 1). In fact, most of the DA in the cerebral cortex is released by NE neurons [53], and extracellular DA is mainly absorbed into the NE neurons through the norepinephrine transporter (NET) [54–56]. Thus depending on the 5-HT receptors involved, an increase in 5-HT levels can either enhance or inhibit DA and NE neurotransmission. An increase in 5-HT levels, can, for instance, enhance 5-HT_2C_R-mediated tonic inhibition of DA and NE neurons, resulting in decreased DA and NE levels, and subsequent inducement or worsening of depressive symptoms.

#### Serotonin-modulated DA and NE inhibition and residual depressive symptoms

Among the prominent underlying mechanisms for TRD, is the proinflammatory cytokine-induced increase in the reuptake, and impairment of the synthesis and release of monoamine neurotransmitters, which results in DA, NE, and 5-HT deficiency [57]. The 5-HT deficiency results in upregulation of inhibitory 5-HT_2C_Rs [48, 58], and thus, an increase in 5-HT levels enhances 5-HT_2C_R-mediated tonic inhibition of DA and NE neurotransmission [48], with a potential for worsened depression symptoms [59]. Indeed, long-term use of SSRIs such as fluoxetine, paroxetine, and sertraline, results in increased 5-HT levels and worsened depression, which is characterized by a significant loss of motivation, reduced appetite, psychomotor retardation, loss of interest, lack of insight, deficits in activities of daily living [36], as well as suicidal thoughts, and akathisia (involuntary severe motor restlessness) [59]. These SSRI-induced depression symptoms are collectively referred to as apathy syndrome or emotional blunting, which is observed in 20 to 92% of individuals who are exclusively treated with SSRIs [60]. These SSRI-induced impairments could potentially be misdiagnosed as residual symptoms or an SSRI-associated treatment-resistant subtype of depression.

The depressive symptoms experienced after prolonged SSRI treatment are observed in enhanced whole brain 5-HT neurotransmission [61], and mainly reflect a sustained reduction in motivational processes, with a generalized suppression of motor output [62]. The underlying mechanisms involve 5-HT-induced suppression of DA [63] and NE [48] release via inhibitory 5-HT_2C_Rs [50, 64] and excitatory 5-HT_2A_Rs on GABA interneurons [64]. These SSRI-induced inhibitory effects on DA and NE neurotransmission and the associated depressive symptoms can be reversed through augmentation of SSRIs with 5-HT_2C_Rs and 5-HT_2A_Rs antagonists, NE reuptake inhibitors, and DAR agonists [64]. This implies that interaction among the three monoaminergic neurotransmitter systems might be an underlying mechanism for residual symptoms or antidepressant treatment resistance (Fig. 1). It also points to the possibility that the time taken to desensitize the 5-HT_2C_Rs and 5-HT_2A_Rs and subsequently facilitate DA and NE neurotransmission, might explain the delayed onset of the therapeutic effects of SSRIs and SNRIs.

#### The dopaminergic system as a convergent mechanism in TRD and antidepressant therapeutic action

The most prominent features of TRD, include anhedonia and motivational deficits, which are associated with a functional deficiency in the dopaminergic reward circuit, comprising of the PFC, nucleus accumbens (NAc), ventral tegmental area (VTA), hippocampus, and amygdala [65, 66]. Targeting the dopaminergic system, in the PFC, hippocampus, NAc, and VTA, produces rapid antidepressant therapeutic effects [67], and there is speculation that disinhibition of the DA neurons via 5-HT_2C_Rs could be an underlying mechanism of action of several antidepressant drugs [48, 63]. This is supported by evidence that the relief of depression symptoms achieved through chronic administration of SSRIs, TCAs, or SNRIs is reversed by low doses of DA receptor (DAR) antagonists [68]. Furthermore, augmentation of antidepressants with pramipexole, a non-ergot D_3_R agonist, seems to be an effective treatment for TRD [69], and compared to SSRIs, SNRIs cause a faster antidepressant effect, which is suppressed by DAR antagonists [70]. This implies a possibility of SNRI-induced increase in DA levels, as an underlying mechanism for the fast antidepressant effect. Indeed, SNRIs such as reboxetine, block NET and elevate NE levels at low doses, but also increase DA levels at higher doses [55, 71, 72]. Based on the compelling evidence of SSRI and SNRI-induced DA release, the reversal of the therapeutic effects of SSRIs, TCAs, and SNRIs through DAR antagonism, and the fast antidepressant effect associated with DAR agonists, it is pluasible to conclude that the dopaminergic system is a convergent mechanism in depression and antidepressant treatment response. The longer duration required for cumulative increase in DA levels might account for the delayed onset of the therapeutic effects of these antidepressants.

#### Dopamine as a modulator of neuroinflammation in TRD

TRD is associated with neuroinflammation, which is believed to play a major role in both the pathogenesis of depression and antidepressant treatment resistance. Systemic pro-inflammatory cytokines leak into the brain through the porous regions of the blood–brain barrier [73], and high concentrations of TNF-α, IL-6, IL-1β, and other pro-inflammatory mediators are associated with decreased DA release [74–77], and reduced effectiveness of antidepressant medications [78–80]. Although DA is primarily synthesized in neurons, it is also synthesized and released by immune cells [81, 82], which also have DA receptors (DARs) and DA transporters (DATs) [83]. This implies that the dopaminergic system can directly modulate the activity of immune cells, which in turn also regulate the DA levels in the brain.

**Fig. 2:**
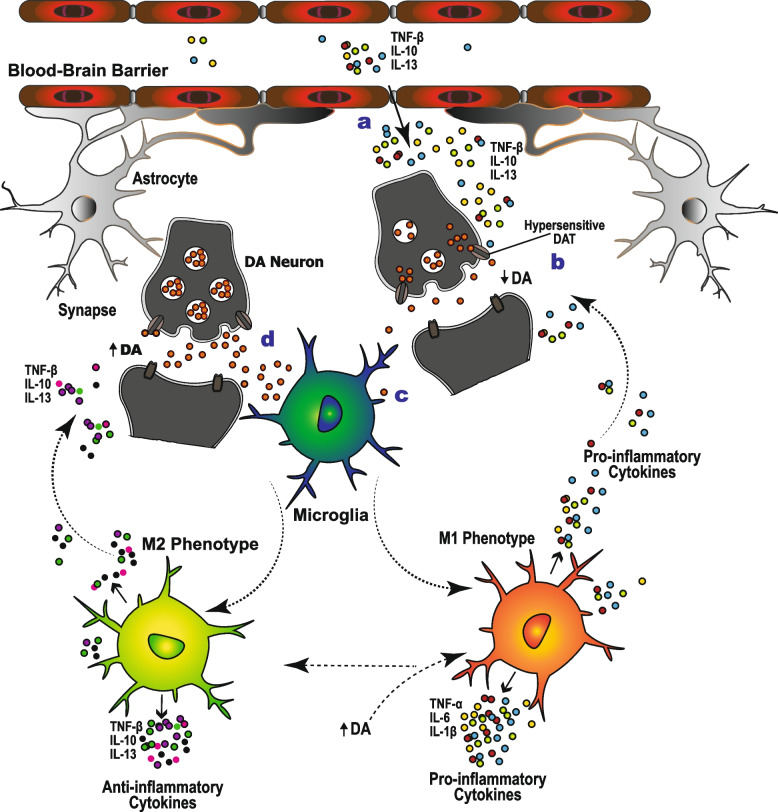
Dopamine as a neuroinflammation moderator in depression. The role of dopamine in depression is not only related to its deficiency but also its involvement in moderation of the neuroinflammatory mechanisms that underlie depression and antidepressant treatment resistance. Pro-inflammatory cytokines such as TNF-α, IL1β, and IL6 leak into the brain through the porous regions of the blood–brain barrier (**a**) and are associated with decreased DA release (**b**). The resulting low levels of DA selectively activate high-affinity D_3_, D_4,_ and D_5_ receptors on microglia (**c**) and convert microglia into the pro-inflammatory M1 subtype, while a high concentration of DA activates the low-affinity D_1_ and D_2_ receptors (**d**), and shifts the microglia into the anti-inflammatory M2 subtype. The pro-inflammatory cytokines released by the M1 subtype induce a DA deficiency, which in turn contributes to an increase in microglial cytokine production, and further heightens DA deficiency, risk of depression, and resistance to antidepressants. An increase in extracellular dopamine levels can result in the conversion of the pro-inflammatory M1 microglia into the anti-inflammatory M2 subtype, and reverse the symptoms

Depending on the DA levels, time of exposure, and their activation state, immune cells express different DAR subtypes [84], which determine the specific effects of DA [81, 82]. For instance, depending on their DAR subtypes, microglia may be activated as the M1 phenotype, which releases pro-inflammatory cytokines, or as the M2 phenotype, which releases anti-inflammatory cytokines [85]. Low DA levels selectively activate high-affinity microglial DARs (D_3_R, D_4_R, and D_5_R), which results in increased release of pro-inflammatory cytokines [86], including TNF-α, IL-6, and IL-1β [76, 77], and is associated with severe depression [87]. Conversely, high DA levels selectively activate low-affinity DARs (D_1_R and D_2_R) and induce anti-inflammatory cytokine release [88, 89]. Bupropion, a DA and NE reuptake inhibitor, increases DA levels and induces a shift from M1 to M2 microglia phenotype, which enhances anti-inflammatory cytokine release and suppresses the release of pro-inflammatory cytokines [90]. A decrease in DA activity is not only associated with increased pro-inflammatory cytokine release [75], but also antidepressant treatment resistance [91], and residual depressive symptoms after prolonged treatment [75]. Indeed, the residual symptoms tend to mainly be motivational and motor in nature, and they are relieved by the augmentation of antidepressants with DA neurotransmission-enhancing agents such as amisulpride [92]. Furthermore, proinflammatory cytokines increase the sensitivity of monoaminergic transporters, including the serotonin transporter (SERT) [93, 94], DAT, and NET [95], which not only increases the uptake and depletion of DA, NE, and 5-HT but also potentially reduces antidepressant treatment response (Fig. 2).

### Neuroinflammation in depression symptom clusters

Depression is characterized by two clusters of symptoms (somatic vs. affective-cognitive) [96], which can be identified using the Beck Depression Inventory-II (BDI-II) [97], and appear to have differential responsiveness to antidepressants. These two clusters of depression symptoms seem to be a consequence of the differential effects of neuroinflammation on the different monoamine neurotransmitter systems.

#### Treatment-responsive depression symptoms

The treatment-responsive subtype of depressive symptoms is psychological (affective-cognitive) and includes sadness, decreased mood, and impaired cognitive functioning [98, 99]. The impairment of cognitive functioning may affect different mental processes including thinking, reasoning, decision-making, learning, memory, attention, and problem-solving [100]. These affective-cognitive symptoms are believed to be a result of inflammation-induced changes in the serotoninergic system [75] and can be alleviated by serotonergic therapies. For example, prolonged treatment with SSRIs increases 5-HT levels and relieves these symptoms, but 20 to 92% of the patients [60], suffer from residual symptoms that mainly include anhedonia, psychomotor retardation, and motivational symptoms [36, 59, 60]. The finding that these residual symptoms are unresponsive to antidepressants that target the 5HT system, implies that different neurotransmitters are involved, and a dysfunction in each of them might underlie particular categories of depression symptoms.

#### Treatment-resistant depression symptoms

The treatment-resistant cluster of symptoms is somatic [99], and it is characterized by fatigue, lack of energy, decreased motivation, motor slowing, reduced appetite, altered sleep [91, 99, 101, 102], anhedonia and other impairments in reward- and motivation-related mechanisms [103]. It is believed that these symptoms may be due to inflammation-induced deficiencies in the dopaminergic [75] and noradrenergic [104] systems. This is supported by evidence indicating that treatment with SSRIs alleviates the affective-cognitive symptoms but has limited or no effect on the somatic impairments [105], that form a set of residual symptoms associated with decreased drive and motivation [104, 106]. On the other hand, SNRIs can increase synaptic NE and DA levels; thereby resulting in fewer residual symptoms, compared to SSRI-based treatments [104]. Indeed, high doses of SNRIs are associated with elevated DA levels [55, 71, 72], and they tend to be more effective than SSRIs [107]; with greater therapeutic effects and better remission rates [108]. Since NE and DA-targeting medications increase dopaminergic neurotransmission, they might be more effective, than 5HT-based therapies, and could be associated with a reduced risk of the residual symptoms observed in patients who receive prolonged SSRI treatment.

#### Molecular mechanisms for cytokine-induced depression symptoms clusters

Through the alteration of kynurenine and tetrahydrobiopterin (BH4) enzymatic pathways, pro-inflammatory cytokines affect the synthesis of monoamine neurotransmitters [109]. Along the kynurenine pathway, pro-inflammatory cytokines activate and upregulate indoleamine 2,3 dioxygenase (IDO) [110], an enzyme that facilitates the conversion of tryptophan into kynurenine instead of serotonin [57, 77, 111]. This reduces the amount of tryptophan available for conversion into 5-hydroxytryptophan (5-HTP), which is a precursor of 5-HT [112]. This implies that the cytokine-induced reduction in tryptophan results in reduced 5-HT synthesis and a subsequent 5-HT deficiency that underlies the pathogenesis of depression and treatment response. Cytokines such as IL-1β and TNF-α also reduce 5-HT synthesis via the inhibition of tryptophan hydroxylase (TPH) mRNA [113], a rate-limiting enzyme involved in the metabolism of 5-HTP into 5-HT [112]. These cytokines also stimulate the serotonin transporter (SERT) via the p38 mitogen-activated protein kinase (p38 MAPK) signaling pathway [114], which increases the uptake of 5-HT [114]; that depletes synaptic 5-HT levels and subsequently contributes to depression symptoms.

**Fig. 3:**
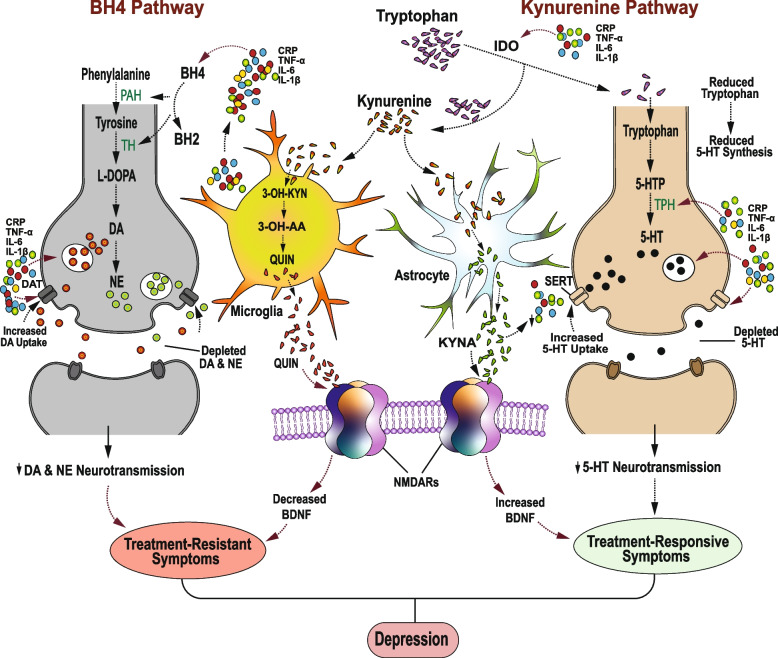
Mechanisms for pro-inflammatory cytokine-induced two-cluster depression symptoms. Pro-inflammatory cytokines interfere with the synthesis of monoamine neurotransmitters through the alteration of kynurenine and tetrahydrobiopterin (BH4) enzymatic pathways. Along the kynurenine pathway, pro-inflammatory cytokines activate and upregulate indoleamine 2,3 dioxygenase (IDO), which facilitates the conversion of tryptophan into kynurenine instead of 5-HTP. This reduces the amount of 5-HTP available for 5-HT synthesis and subsequently results in a 5-HT deficiency, which is associated with treatment-responsive cognitive symptoms of depression. The diverted kynurenine is converted into kynurenic acid (KYNA) by astrocytes, and into quinolinic acid (QUIN) by microglia. Through the activation of NMDARs, QUIN induces neurotoxic effects, including reduced BDNF synthesis. An increase in QUIN synthesis is associated with a corresponding decrease in both KYNA levels and BDNF synthesis and an increase in the release of pro-inflammatory cytokines, which are linked to decreased responsiveness to antidepressant treatment. KYNA is an NMDAR antagonist that induces neuroprotective processes, including BDNF synthesis and attenuation of pro-inflammatory cytokine levels, which are associated with enhanced responsiveness to antidepressants. Along the tetrahydrobiopterin (BH4) pathway, pro-inflammatory cytokines induce oxidative depletion of BH4, and a subsequent reduction in the levels of the BH4-dependent enzymes involved in DA and NE synthesis. The resulting DA and NE deficiency is associated with treatment-resistant somatic symptoms

Within the astrocytes and microglia, kynurenine is respectively converted into kynurenic acid (KYNA) and quinolinic acid (QUIN) [109, 115, 116], which are neuroactive glutamatergic metabolites [109]. KYNA acts on both N-methyl-D-aspartate (NMDA) and α7-nicotinic acetylcholine (α7-nAChR) receptors to induce neuroprotective effects [109]. KYNA-induced neuroprotective effects include suppression of pro-inflammatory cytokine [119] and excessive glutamate [117] release, enhanced astrocytic uptake of extracellular glutamate [118], and increased brain-derived neurotrophic factor (BDNF) synthesis [119]. KYNA also regulates glutamate, DA, and GABA concentrations [120, 121], which along with its anti-inflammatory properties and inducement of BDNF synthesis [119], could be underlying mechanisms for KYNA-induced antidepressant [120, 122], and treatment response enhancement [120, 123] effects. On the other hand, QUIN is an NMDA receptor agonist [116] that induces neurotoxic effects, including promotion of oxidative stress, apoptosis [110], reduced BDNF levels [124, 125], and degeneration of glutamatergic and dopaminergic neurons [125], which are linked to antidepressant treatment resistance. An increase in QUIN synthesis is associated with a corresponding decrease in the production of neuroprotective KYNA [126], and an increase in the release of pro-inflammatory mediators, including CRP [127], TNF-α and IL-6 [128], which are associated with decreased responsiveness to antidepressant treatment [79] (Fig. 3). Indeed, ketamine, an efficacious novel treatment for TRD, is associated with increased KYNA and BDNF synthesis, and attenuation of both QUIN and pro-inflammatory cytokines levels [32]. A decrease in KYNA to QUIN ratio is associated with increased depressive episodes, and anhedonia [129], which is among the typical treatment-resistant symptoms. This suggests that a reduction in KYNA, and excessive QUIN synthesis, may contribute to increased vulnerability to depression and treatment-resistant depressive symptoms.

The alteration of the tetrahydrobiopterin (BH4) pathway involves pro-inflammatory cytokine-induced activation of GTP-cyclohydrolase 1 (GTP-CH1) [130], which is an initial and rate-limiting enzyme in the formation of BH4 [130], an enzyme cofactor involved in the biosynthesis of 5-HT, DA, and NE [57, 131]. The activation of GTP-CH1 results in an upregulation of the synthesis of these monoamines [132], which seems to occur during acute, but not chronic, inflammation. For example, acute inflammation triggers upregulation of BH4 levels and increased DA synthesis, while chronic inflammation induces oxidative decrease in BH4 and subsequent impairment of DA synthesis [89, 131]. Pro-inflammatory cytokines increase oxidative stress through the generation of reactive oxygen species (ROS), and reactive nitrogen species (RNS), which contribute to the oxidation and depletion of BH4 [98, 133]. In turn, the depletion of BH4 disrupts the activity of nitric oxide synthase (NOS) enzymes, which catalyze the formation of nitric oxide (NO) via the oxidation of L-arginine, as well as several BH4-dependent enzymes involved in DA and NE synthesis [132]. In particular, the enzyme, phenylalanine 4-hydroxylase (PAH) catalyzes the conversion of phenylalanine to tyrosine [134], while tyrosine 5-hydroxylase (TH) catalyzes the conversion of tyrosine into L-DOPA the precursor of DA [75], that is in turn a precursor of NE [131]. Thus, the cytokine-induced decrease in BH4 levels impairs DA synthesis and results in the depletion of both DA and NE, which is an underlying mechanism for motivational deficits and other treatment-resistant depression symptoms.

Furthermore, pro-inflammatory cytokines such as IL-1 β, and TNF-α suppress the expression of the vesicular monoamine transporter 2 (VMAT2), which interferes with the packaging of DA into secretory vesicles and consequently reduces the amount of presynaptic DA [82]. There is also evidence that these cytokines decrease the levels of monoaminergic neurotransmitters, through increased expression of sensitive 5-HT, NE, and DA transporters [115]; thereby resulting in a high rate of monoamine uptake and a deficiency in extracellular NE, 5-HT, and DA. In a recent study, infusion of IL-6 reduced rodent striatal DA levels by 40% within 30 min [135], which was too rapid and massive to be an exclusive result of cytokine-induced decrease in DA synthesis, and thus implying a potential involvement of cytokine-mediated increase in DA uptake [134]. Indeed, pro-inflammatory cytokines such as TNF-α increase SERT and DAT activity and induce depressive symptoms [136], while on the other hand, SSRIs block SERT and prevent TNF-α-induced depressive-like symptoms [137]. Evidence suggests that the cytokine-induced dysfunction of the serotoninergic system is associated with the treatment-responsive cluster of depressive symptoms, while treatment-resistant symptoms involve dysfunction of the dopaminergic [75] and noradrenergic [104] systems (Fig. 3).

### Role of stress in neuroinflammation and TRD

The link between stress and TRD is complex, but recent evidence indicates that both the pathogenesis of depression and unresponsiveness to treatment, may be due to prolonged cortisol release [138] that downregulates glucocorticoid receptors (GRs) on the neurons of the hypothalamus and pituitary, and results in dysregulation of the hypothalamic–pituitary–adrenal (HPA) axis.

#### Stress and HPA axis dysregulation in pro-inflammatory cytokine release

**Fig. 4:**
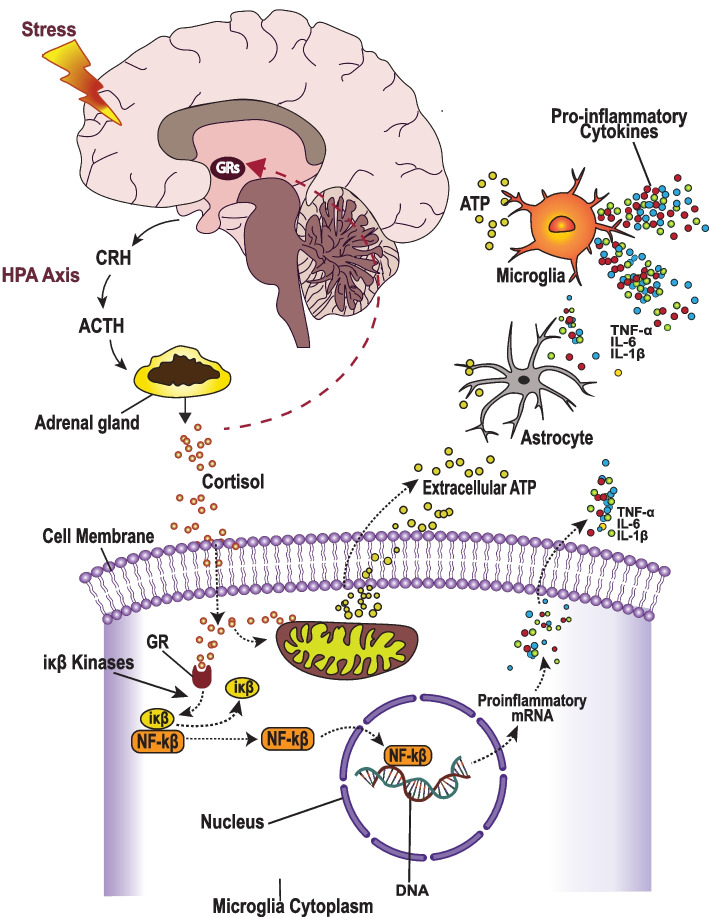
Mechanisms for stress-induced neuroinflammation and depressive symptoms. Stress-induced excessive cortisol release induces a downregulation of glucocorticoid receptors (GRs), which results in dysregulation of the HPA axis, and impairment of the negative feedback loop that normally regulates cortisol levels. The excessive cortisol binds the GRs in microglia and results in transcription of pro-inflammatory cytokines, and increased release of ATP into the extracellular space. When ATP binds P2X_7_Rs on astrocytes and microglia it induces the release of interleukin-1 β (IL-1β), which triggers the release of other pro-inflammatory mediators, that contribute to depression and impaired treatment responsiveness

Chronic stress induces excessive cortisol release, which results in adaptive downregulation of GRs and subsequent desensitization of the HPA axis [139]. The desensitization of the HPA axis impairs the negative feedback loop, which results in a sustained stress response [140], that consequently contributes to neuroinflammation, depression [141], and antidepressant treatment resistance [142]. Under normal circumstances, the binding of GRs by cortisol promotes the transcription of anti-inflammatory proteins like Ikappaβ kinase (Iκβ or IKK) [143], which inhibit the synthesis of pro-inflammatory cytokines via the inactivation of proinflammatory transcription factors, such as the inflammatory nuclear factor (NF-κB) [141, 143]. On the other hand, excessive cortisol impairs the transcription of anti-inflammatory proteins, and instead promotes an increase in pro-inflammatory gene transcription and release of pro-inflammatory cytokines such as IL-6, IL-1, and TNF-α [143] by activated microglia [144] (Fig. 4). This is observed among TRD patients, who when compared to healthy controls, tend to have lower levels of anti-inflammatory mediators such as IL-4, Transforming Growth Factor beta (TGF-β1)[145] and miR-146a-5p [146], and instead have higher concentrations of pro-inflammatory mediators such as IL-1β, IL-2, IL-6, IL-12, Monocyte Chemoattractant Protein (MCP) − 1, and C-reactive protein (CRP) [145, 146]. The increase in these pro-inflammatory mediators is associated with impairment of uptake of glutamate by astrocytes [147] and subsequent glutamate/GABA imbalance and monoaminergic dysfunction, that characterize TRD.

#### Stress-activated ATP in neuroinflammation and antidepressant treatment resistance

The underlying mechanisms for pro-inflammatory cytokine release involve chronic stress-induced release of adenosine 5’-triphosphate (ATP) from microglia [148]. An increase in extracellular concentrations of ATP, which is an endogenous danger signal, activates purinergic P2X_7_ receptors (P2X_7_Rs) on astrocytes and microglia [149]. The activation of P2X_7_Rs triggers release of interleukin-1 β (IL-1β), which in turn induces a neuroinflammatory response [150] (Fig. 4). Evidence shows that single-nucleotide polymorphisms (SNPs) in rs2230912 and rs1718119 are associated with a human P2X_7_R variant, characterized by increased sensitivity to ATP, IL-1β release [151], and more severe depression [152]. Evidence also shows that antagonism of P2X_7_R relieves depressive symptoms, and genetic deletion of P2X_7_R in mice results in a depression-resistant phenotype [149] characterized by the absence of IL-1β release. Interestingly, neuroinflammatory responses are suppressed by P2X_7_R antagonists, inhibitors of stress-activated protein kinases [153], and pretreatment with astrocyte toxin, L-α-aminoadipate (L-AAA) [154]. These findings suggest that stress-induced ATP release, the activation of P2X_7_Rs, and the subsequent release of pro-inflammatory cytokines play a major role in the etiology of depression and antidepressant treatment resistance.

### Role of GABA and glutamate in TRD

There is accumulating evidence showing that the pathological mechanisms of TRD involve complex interactions among γ-aminobutyric acid (GABA), glutamate, DA, NE, and 5-HT neurotransmitter systems. Glutamate, DA, and NE are excitatory, while GABA and 5-HT are inhibitory neurotransmitters [65]. The balance between the inhibitory GABAergic and excitatory glutamatergic neurotransmitter systems [25] is very essential in brain functioning since all neurons and glial cells have glutamate and GABA receptors [155]. Glutamate is the precursor of GABA [156], and the balance between these two neurotransmitters is entirely dependent on astrocytes, which uptake most of the glutamate from the extracellular space, and convert it into GABA using an enzyme, glutamic acid decarboxylase (GAD) [147]. Some of the glutamate is converted into glutamine with the aid of glutamine synthetase, which is exclusively found in astrocytes [156]. The glutamine released into the extracellular space is absorbed by both the GABAergic and glutamatergic neurons and used to replenish their respective neurotransmitters [157, 158]. The balance between excitatory glutamatergic and inhibitory GABAergic systems is very essential in the modulation of a complex interaction among the monoaminergic neurotransmitter systems, which contributes to the pathogenesis of depression and antidepressant treatment response.

#### GABA/Glutamate imbalance in TRD

**Fig. 5:**
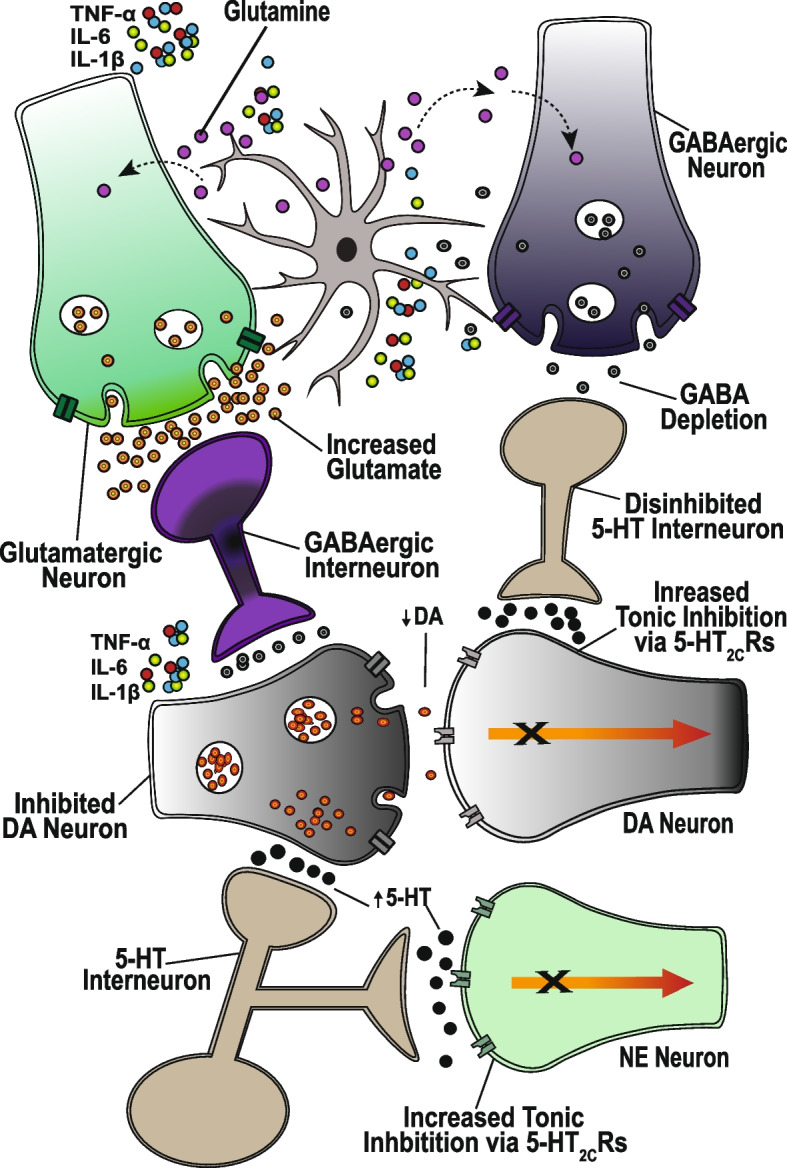
Astrocyte dysfunction-mediated glutamate/GABA imbalance in TRD. Pro-inflammatory cytokines such as IL-1β, IL-2, IL-6, and IL-12 induce astrocytic dysfunction, which results in decreased glutamate uptake, reduced glutamine synthesis, and subsequent increase in extracellular glutamate, and reduced GABA levels. The decrease in GABA is associated with reduced inhibitory neural mechanisms, including the disinhibition of 5-HT and GABA-mediated tonic inhibition of the dopaminergic pathways. This results in decreased DA release in the brain pathways involved in the regulation of reward and motivation, and consequently contributes to treatment-resistant or residual symptoms. The excessive glutamate levels overstimulate NMDARs and result in neurodegeneration and decreased BDNF levels, which are also associated with treatment resistance. Evidence suggests that BDNF adaptations and associated synaptic plasticity are required in antidepressant response and could be an underlying mechanism for the delayed therapeutic effect of some antidepressants

Among the prominent underlying mechanisms for TRD, is the dysregulation of astrocytic functionality, which results in a GABA/glutamate imbalance. For example, pro-inflammatory mediators activate microglia and suppress the astrocytes’ ability to clear extracellular glutamate [147]. The excessive extracellular glutamate associated with astrocytic dysfunction overstimulates NMDA receptors [147]. This over-activation of NMDARs induces suppression of the synthesis and release of BDNF, which results in reduced neuroplasticity and structural alterations that underlie the pathogenesis of depression and responsiveness to antidepressant medication [159]. On the other hand, the impairment of astrocytic absorption of glutamate can result in GABA deficiency, since glutamate is the precursor of GABA [156, 160], and the astrocytic conversion of glutamate into glutamine is a crucial step in the replenishment of both neurotransmitters [157, 158, 161]. The depletion of GABA can result in the disinhibition of various inhibitory mechanisms such as the 5-HT and GABA-mediated tonic inhibition of DA and NE neurotransmission and subsequently contribute to depressive symptoms. For example, a decrease in GABA levels in the Parvalbumin (PV), a convergent brain region for projections involved in the regulation of mood [162] and motivation [163], results in the disinhibition of GABA to GABAergic projections to the VTA, and increase in VTA GABA release, and inhibition of dopaminergic neurotransmission [164]. The increased inhibition of dopaminergic neurons, results in decreased DA release and subsequent anhedonia and other motivational symptoms [24] that characterize TRD. The potential underlying mechanisms for the astrocytic dysfunction-mediated glutamate/GABA imbalance are illustrated in Fig. 5.

#### Glutamate and BDNF in antidepressant treatment resistance

Excessive glutamate in the extrasynaptic space induces different effects depending on whether it binds α-amino-3-hydroxy-5-methyl-4-isoxazolepropionic acid (AMPA), NMDA, or Kainite receptors. Of particular interest is the observation that activation of extrasynaptic AMPARs increases BDNF levels, while NMDAR activation results in inhibition of the synthesis and release of BDNF [147]. According to the neurotrophic hypothesis, the underlying mechanism for the pathogenesis of depression is BDNF deficiency [6, 165], and evidence shows that TRD patients tend to have a greater decrease in BDNF expression levels compared to patients who are responsive to antidepressants [166, 167]. BDNF is involved in Hebbian plasticity, which is a positive feedback mechanism that occurs within seconds or minutes, when dendrites are stimulated via AMPARs [168] and/or NMDARs [169]. Continuous stimulation of dendrites by the same signals induces long-term potentiation (LTP) [170], which is a prolonged increase in synaptic strength, characterized by BDNF-modulated rapid increase in NMDAR [169] or AMPAR expression [171–173], thickening of the postsynaptic density protein, enlargement of dendritic spines, and subsequent increase in signal transmission efficiency [168]. This implies that the reduction in BDNF observed during depression, and the associated impairment of LTP and other forms of synaptic plasticity may reduce responsiveness to signals, including those induced by antidepressants.

Indeed, signaling via BDNF and its high-affinity tyrosine receptor kinase B (TrkB) [171] is required in both LTP and antidepressant response [170]. Evidence shows that a reduction in BDNF impairs [168, 174, 175], while an increase in BDNF improves the effectiveness of antidepressants [176]. Prolonged use of antidepressant therapy, including SSRIs, TCAs, and electroconvulsive therapy (ECT), increases BDNF expression [177], and infusion of BDNF into brain regions involved in depression, induces antidepressant effects [178, 179]. This suggests that an increase in BDNF-TrkB signaling is required in antidepressant treatment response, and the BDNF-related adaptations following prolonged use of antidepressants are consistent with the duration of weeks or months required for the conventional antidepressants to induce therapeutic effects [180]. This implies that delayed or impaired BDNF-TrkB signaling-related adaptations may be an underlying mechanism for the lack of antidepressant treatment response observed in TRD.

### Genetics vulnerability to depression and treatment resistance

Depression is a heterogeneous polygenic trait, whose heritability is in the range of 20–50% [165, 181], but the identification of the genes that account for particular depression subtypes and variation in treatment response is still a major challenge. A polygenic trait such as depression, is determined by thousands of genetic variants and can be influenced by the environment and other factors. This complicates the identification of specific genetic biomarkers for detecting individuals who might be at high risk of TRD [182]. Indeed, currently, there are no confirmed genetic biomarkers for TRD, but there are some genes, that show replicated association and they are linked to synaptic plasticity, and neurotransmitter systems [182].

#### Genetic biomarkers for TRD

Whereas polygenic traits such as depression have a lot of variability, which complicates the replication of previous research findings, there are some genetic markers, that seem consistent in TRD. For example, within the glutamatergic neurotransmitter system, increased risk of TRD is associated with rs12800734, rs1954787 [183] and rs11218030 [184] variants of the GRIK4 gene, which encodes a kainate receptor subunit, as well as rs890 and rs1805502 variants of the GRIN2B gene [185], that encodes the NR2B subunit of NMDAR [185, 186]. Furthermore, recent evidence shows that TRD is associated with a decrease in BDNF levels, and BDNF Val66Met polymorphism is associated with resistance to SSRIs [167]. Within the serotonergic system, antidepressant resistance is associated with polymorphisms in the 5HT_2A_R [187], and a serotonin transporter gene (5-HTTLPR/SLC6A4) [183]. A study by the European Group for the Study of Resistant Depression (GSRD) involving 2762 patients in eight European countries found single nucleoid polymorphisms (SNPs) within the GAP43, ITGB3, PPP3CC, ST8SIA2, and CHL1 genes to be linked to TRD [14]. However, the results are still inconsistent, and very few of the TRD genetic associations have been consistently replicated. The polygenic nature of depression is expected to create many genetic variations, and consistent identification of depression-related genes requires very large population samples. More research focusing on large depressed populations could reveal more specific genetic biomarkers linked to the pathogenesis of depression and antidepressant treatment response.

#### Epigenetics of the human endogenous retroviruses

Environmental risk factors such as childhood adversity or life stressful events [188–190], can interact with genetic predispositions in the etiology of depression and antidepressant treatment response. For instance, human endogenous retroviruses (HERVs), which are ancient evolutionary retrovirus RNA insertions in approximately 8% of modern humans’ DNA [191–193], are typically epigenetically repressed but can be reactivated by intrinsic or extrinsic factors [192], such as infections, inflammation, antipsychotic drugs [194], and stressful stimuli [195, 196]. Although they do not have infectious viral particles, some HERVs retain the ability to encode and release transcripts and proteins [197], which can contribute to the pathogenesis of mental illness. Canli (2019) proposed that individual differences in vulnerability and resilience to psychopathology may derive from the polymorphic structure of HERVs, as well as the balance of activated pathogenic versus protective HERVs [198]. For example, compared to healthy controls, the expression of potentially protective GAG proteins encoded by HERV-W is significantly reduced in the brains of individuals with depression as well as those with schizophrenia, and bipolar disorder [196]. A recent study found the HERV-W protein expression was higher in mental disorder patients compared to controls, and higher levels of HERV-W protein expression were associated with lower concentrations of an anti-inflammatory cytokine (IL-10), but increased pro-inflammatory cytokines, TNF-α and IFN-γ, in bipolar depression patients (Rangel et al., 2024). Furthermore, genetic data from 792 post-mortem brain samples, revealed 9 HERV signatures for depression, including five and two on chromosomes 1p31, and 9p23 respectively, while chromosomes 3p21 and 14q24, each had one HERV signature linked to depression [199]. In the same study, multiple expressions on chromosome 1p31 correlated with increased risk, but ERVLE_1p31.1c was independently associated with depression. These results suggest that HERVs are associated with increased inflammation and genetic vulnerability to bipolar disorder, schizophrenia, depression, and possibly other mental disorders.

**Fig. 6:**
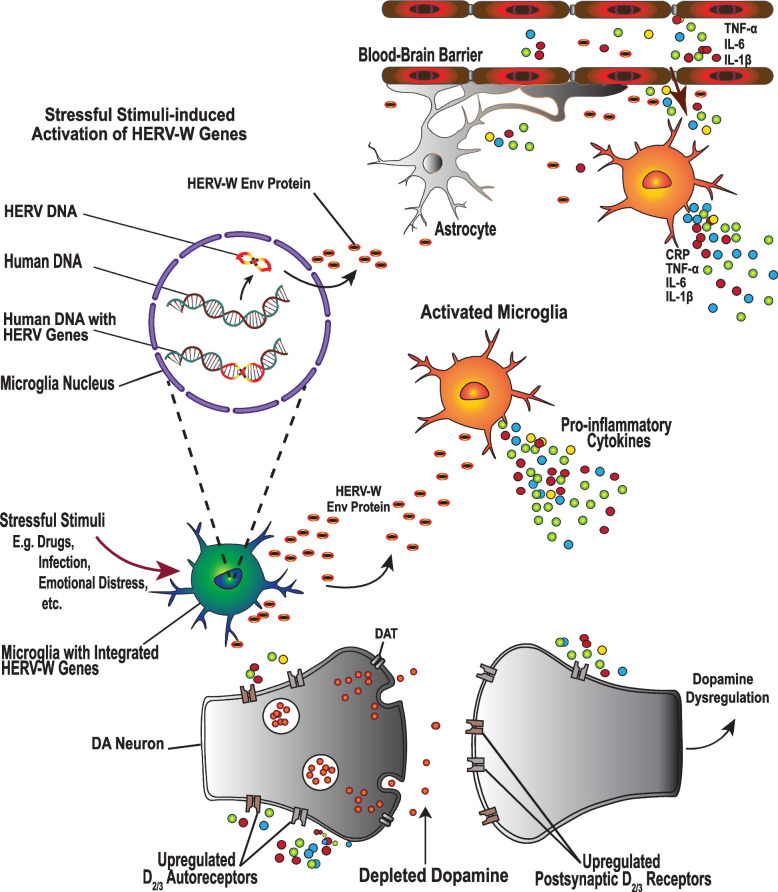
Human endogenous retroviruses (HERVs) as regulators of Dopamine and Neuroinflammation in Depression. Although the HERV genetic insertions in the human genome are typically silenced and not pathogenic, they are activated by intrinsic and extrinsic stressors, such as infections, inflammation, and drugs. Once activated, they induce the release of pro-inflammatory cytokines, including IL-1β, IL-6, TNF-α and IFN-γ by both human microglia and astrocytes, and are associated with dysregulation of the blood–brain barrier [201], which increases its permeability to peripheral immune cells, and inflammatory mediators. HERV proteins are over-expressed in the brain tissue of individuals with psychopathological conditions and are not only associated with neuroinflammation but also increased expression of pre-synaptic and post-synaptic D_2/3_Rs, which are associated with disruption of dopaminergic transmission that characterizes depression and variation in treatment response

Although a direct link between HERVs and depression is still unclear, HERV-W-env protein expression is associated with increased release of pro-inflammatory cytokines [200], including IL-6, IL-8 [201], IL-1β [193, 202], and TNF-α and IFN-γ [200], which are associated with BDNF reduction [179], HPA axis dysfunction [203], depletion of monoamine neurotransmitters [75, 104], and other pathological mechanisms of depression. Furthermore, recent evidence shows that among schizophrenic patients, HERV-W is associated with significantly higher expressions of both DA receptors, D_2_R [204], and D_3_R [205] compared to controls. Over-expression of D_2_R and D_3_R receptors is also observed in depression, with both *Presynaptic* D_2_/D_3_ autoreceptors [206, 207], and *postsynaptic* D_2_/D_3_ receptors inhibiting DA release [206, 208], which can potentially contribute to treatment resistance. Thus, whereas the specific mechanisms of HERVs in the context of depression remain to be investigated, the finding that they are associated with increased release of pro-inflammatory cytokines, and increased expression of D_2_R and D_3_R, converges to suggest a potential link between HERVs, depression pathogenesis, and antidepressant treatment resistance (Fig. 6).

## Management of treatment-resistant depression

Consistent evidence shows that less than 33% of patients achieve remission with the first-line antidepressant treatment [209, 210], implying an approximate 66% non-recovery rate and about 30% do not benefit from sequential antidepressant treatments [31]. The management of treatment-resistant depression is thus still a major challenge, and given the heterogeneous nature of this disorder, there is no consensus on the best treatment approach [44]. This mainly arises from the absence of a universally agreed upon TRD definition, that would be a basis for an accurate diagnosis, and choice of a treatment approach. The most widely adopted definition, and the basis of several treatment guidelines [211], is that TRD is a subtype of depression, characterized by an inadequate response to at least two antidepressants of adequate trial dosage and duration, and following the required level of adherence to the treatment.

There are five TRD staging models, including: 1) the Maudsley Staging Model (MSM); 2) Thase and Rush Model; 3) Antidepressant Treatment History Form; 4) Massachusetts General Hospital Staging model; and 5) European Staging Model, which are used to determine the adequacy of prior antidepressant treatment trials, classify the patients’ level of treatment resistance, predict the potential antidepressant therapy success, and guide in the selection of treatment strategy [212]. However, these staging models do not have proven predictive validity of treatment success and are not routinely used. The choice of a treatment strategy largely depends on the patients’ response, with partial responders (25–50% improvement) and non-responders (< 25% improvement) [44], requiring different antidepressant treatment strategies [42, 44, 213]. The management of TRD typically involves the use of different treatment strategies, including pharmacotherapy, somatic therapies, psychotherapy, and various other novel trial-and-error strategies, which are often used in combination with conventional strategies.

### Pharmacological approaches

When managing TRD the common assumption among clinicians is that nonresponse to treatment is due to having not used an adequate dose, a correct type, and/or combination of antidepressant medications. Most clinicians use a trial-and-error approach, and the treatment strategy may involve antidepressant dose optimization, switching, combination, and/or augmentation with other adjunctive drugs or agents [210].

#### Switching and augmentation

Among the most commonly utilized TRD treatment approaches, is switching to bupropion, an NE-DA reuptake inhibitor, and augmentation with either bupropion or aripiprazole, a partial DA agonist [209]. Switching may have the disadvantage of losing the partial efficacy of the current antidepressant [214] and it is thus recommended that partial responders (with a 26–49% symptom reduction) first use augmentation with an antipsychotic drug to potentiate the effectiveness of the currently used antidepressant [44, 214]. Whereas there is limited evidence of the therapeutic advantage of one approach over the other, augmentation with antipsychotics is reported to be more efficacious than switching antidepressants [44, 209]. Indeed, results of clinical trials indicate that compared to antidepressant monotherapy, augmentation with atypical antipsychotics is more efficacious for TRD patients with a high level of treatment resistance [215]. A recent study revealed that the most common TRD treatment approach was augmentation (71%), mainly with antipsychotics, compared to switching from one antidepressant to another (29%) [44]. The finding that augmentation with antipsychotics (DA agonists) is more efficacious than switching, could further underscore the involvement of the dopaminergic system in TRD, and this treatment strategy could also be effective for SSRI-induced anhedonia and motivational symptoms.

##### Switching guidelines

Switching is recommended as the first strategy for non-responders (with less than 25% symptom reduction) since the current antidepressant is already ineffective. However, switching should be done after confirming nonresponse to an optimal dose of the first antidepressant, to which a patient has adequately adhered, for an optimal duration [216] of 6–8 weeks or longer [217]. An advantage of switching is that it is less likely to induce the adverse side effects associated with augmentation drug interactions [5, 218]. However, a rapid change from one antidepressant to another without allowing for a wash-out period can also cause drug interactions [219] and subsequent drug toxicity conditions, like serotonin syndrome [220]. A conservative switching approach is thus recommended, to ensure that before a new antidepressant is started, the body does not have a significant concentration of the previously used medication [220]. This can be achieved through a gradual reduction in the dose (tapering) of the current antidepressant, which may take several days or weeks, followed by a medication-free period that allows for its elimination from the body [220]. Depending on factors such as the patient’s condition, antidepressant type, and rate of drug metabolism, this tapering period may greatly vary; lasting for days, weeks, or months for some patients.

##### Limitations of switching

Whereas the gradual dose reduction ensures that the patient does not experience withdrawal symptoms, the period of abstinence from antidepressants, which typically ranges between at least 4 weeks to 6 months [221], can contribute to relapse or worsening of the symptoms [219, 220]. This is further complicated by the fact that the current clinical practice guidelines do not offer any guidance on dose adjustments, and how to differentiate and/or manage the withdrawal and relapse symptoms [221]. This is not only a challenge to the clinicians who have to operate at their discretion, but also to the patients whose symptoms and concerns may not be adequately addressed.

Another issue of concern is that switching antidepressants is effective for only about 25% of the patients [222], and there is no evidence that the commonly recommended switching between antidepressant classes could be more effective than switching within an antidepressant class [223]. Furthermore, recent evidence suggests that about 20% of the patients who do not respond to treatment during the first 4 weeks, can respond during a 5 to 8-week period, while about 10% can respond between 9 to 12 weeks [42]. This suggests that switching antidepressants can result in the loss of the therapeutic benefits of the initially used class of antidepressants, whose potential therapeutic efficacy would be realized with prolonged use. Furthermore, switching between antidepressant classes has a potential danger of increased risk of adverse side effects among patients who may not have been affected by the initial class of antidepressants. Thus, more research is needed to assess whether there might be significant short- and long-term therapeutic benefits of staying within a given class or switching between antidepressant classes.

### Non-pharmacological approaches

Due to the increased realization that conventional pharmacotherapies for depression are not effective and are associated with adverse side effects, there is a growing interest in the use of non-pharmacological therapies. There are various kinds of therapies being used across the world, but here we mainly present the evidence-based approaches, whose therapeutic effects to some extent compare with convention pharmacotherapies. These non-pharmacological approaches include psychotherapy, and various somatic or neurostimulation therapies.

#### Psychotherapy

Although not often given as much as attention as pharmacological and somatic interventions, the ever-increasing evidence of the pathological role of stress-induced dysregulation of the HPA axis in TRD underscores the value of psychotherapy. Indeed, the results of a recent network meta-analysis that considered 101 randomized clinical trials (RTCs), revealed that for both chronic and treatment-resistant depression, psychotherapy is as effective as antidepressant medication, and using it in combination with antidepressants yields significantly greater therapeutic outcomes than independent use of either therapeutic modalities [224]. Another meta-analysis of RCTs revealed that psychotherapy is effective for the treatment of non-responders with a medium to large effect size, and the effects were still observable at follow-up [225]. A meta-analysis of 21 RCTs assessing the effectiveness of seven different psychotherapies revealed that the most frequently used psychotherapies were cognitive behavior therapy, mindfulness-based cognitive therapy, and interpersonal psychotherapy, and they were all associated with better therapeutic outcomes for TRD if used as an add-on to the usual pharmacotherapy or clinical management [226]. Furthermore, the majority of depressed patients have a preference for psychotherapy over antidepressant medication, and those treated in accordance with their preferences show better therapeutic outcomes [226].

However, despite its recognized effectiveness and preference by the patients [226], the current definitions of TRD do not include psychotherapy [40]. It is evident from the results of RCTs that whether used exclusively or in combination with antidepressants or other somatic therapies, psychotherapy is effective for TRD. We thus, recommend more high-quality RCTs and meta-analyses to further validate its efficacy, and for the TRD definition and guidelines to include psychotherapy as an augmentation to antidepressants and other treatment modalities.

### Neurostimulation therapies

Recent developments in the management of TRD include the use of various techniques such as electromagnetic fields, neural implants, or clinically appropriate electric currents to stimulate the brain or other parts of the nervous system. Whereas some of these approaches are still being studied, there are a number of them, which are of proven clinical significance and approved for TRD management. The commonly used techniques include Transcranial Magnetic Stimulation (TMS), Electroconvulsive Therapy (ECT), and Vagus Nerve Stimulation (VNS).

#### Transcranial Magnetic Stimulation (TMS)

TMS is a noninvasive procedure in which an electromagnetic coil is placed on the scalp and used to stimulate different brain regions, especially the prefrontal dorsolateral cortex (PFDLC) [146, 227]. The FDA-approved TMS procedure for TRD involves daily and repeated prefrontal cortex stimulation for 4 to 6 weeks [228]. Among the commonly used neurostimulation therapies for TRD is Repetitive TMS (rTMS) [146], which typically involves five daily, repetitive stimulations of the brain, for a total of about 20 to 30 sessions by the end of the treatment period [228]. TMS is a safe and efficacious TRD treatment, and a recent study found a response of 46.3% and a remission rate of 33.6% [229]. However, TMS therapeutic mechanisms, are still unknown, and the clinical outcomes differ from one TRD patient to another, even when the same procedures are followed [146]. This finding might further demonstrate the heterogeneous nature of TRD, characterized by variability in the pathological mechanisms, which require individualized treatment. Further research is needed to identify the underlying therapeutic mechanism, the reasons for the variability in response, and the potential long-term side effects of repeated stimulation of the brain.

#### Electroconvulsive Therapy (ECT)

ECT is believed to be one of the most efficacious and gold-standard therapies for TRD [230]. Recent data suggests that ECT is associated with an antidepressant response of 56–85% and a remission rate of about 48% among TRD patients, and the overall response is comparable to that of antidepressant drugs and psychotherapy for non-TRD patients [211]. Interestingly, evidence shows that compared to the 13% remission rate achieved with conventional antidepressants, ECT is associated with remission rates that tend to be in the range of 50 –70% in TRD [230]. However, even though ECT appears to be more efficacious than most other TRD therapies, the level of acceptability and utilization by patients is still low. The low acceptability of ECT might stem from the adverse side effects associated with this technique. For example, results of meta-analyses indicate that ECT causes cognitive dysfunction, including immediate impairment of learning and memory, that lasts for about 14 days after the therapy [231]. However, in some cases these ECT-induced impairments can last for weeks or months [232], and some patients may experience temporary confusion and seizures [233]. Thus, whereas ECT is an effective antidepressant therapy, it needs to only be administered in situations where the therapeutic benefits outweigh the adverse side effects.

#### Vagus Nerve Stimulation (VNS)

Vagus nerve stimulation is a neuromodulation technique, which was approved by the FDA as an adjunctive therapy for TRD [234], and it involves electrical stimulation of the vagus nerve, using a surgically implanted pulse generation device [235]. There is, however, a recently developed noninvasive transcutaneous vagus nerve stimulation (tVNS), which is increasingly becoming popular [236]. The vagus nerve (10th cranial nerve), extends to different organs, and it is involved in the regulation of various autonomic functions, including the immune response and release of neurotransmitters associated with depression. Stimulation of the vagus nerve results in the activation of the nucleus of the solitary tract (NTS) in the brainstem, which in turn stimulates various brain regions, including the main noradrenergic center (locus coeruleus), that has projections to the amygdala, dorsal raphe nucleus, hippocampus, and other limbic and cortical structures [237] involved in emotional regulation, and implicated in depression [238].

A recent study found that VNS has a significant long-term therapeutic efficacy, with a mean depression severity reduction score of 59.9% and a response rate of 87% [234]. Furthermore, a long naturalistic study of the efficacy of VNS as an adjunctive therapy for TRD revealed a 67.6% cumulative response rate over a 5-year period, which was significantly higher than the 40.9% response rate for the treatment-as-usual TRD patients [239]. In a related study, TRD patients who received adjunctive VNS had a significantly better quality of life compared to the treatment-as-usual group, which was observed starting at 3 months and sustained for a 5-year period [240]. Although the underlying mechanisms are not fully known, stimulation of the vagus nerve induces an increase in the release of GABA, which is essential in neural plasticity [236, 238], and also alters the levels of glutamate, 5-HT, DA, and NE [237]. Evidence indicates that VNS increases 5-HT, NE, and BDNF levels, and lowers pro-inflammatory biomarkers, including IL-1β, IL-6, TNFα [241], and IL-7, as well as other inflammation mediators such as vascular endothelial growth factor C (VEGFc), VEGF receptor-1 (Flt-1), Chemokine (C–X–C motif) ligand (CXCL) 2, CXCL8, CCL13, CCL17, and CCL22 [234], which are implicated in TRD. Overall, VNS is efficacious for TRD and has long-term therapeutic effects, which could be a consequence of vagus nerve stimulation-induced suppression of neuroinflammation, as well as increased BDNF, and release of monoamines and other neurotransmitters involved in depression.

## Emerging therapies and future directions

Since both the conventional and recently approved antidepressant therapies are not effective for many patients, more novel therapeutic approaches are needed. Currently, there are several ongoing Phase I–III clinical trials investigating the efficacy of novel therapeutic agents for TRD, which include Ketamine, Psychedelics, anti-inflammatory mediators, as well as antagonists and/or agonists for DA, NMDA, metabotropic glutamate receptor (mGlu5), GABA, and opioid receptors [242]. Some of these emerging therapies have been approved for use in some countries and studies about their mechanisms of action, therapeutic efficacy and tolerability are still ongoing.

### Ketamine as a TRD therapeutic agent

Among the recently discovered, and the first to be approved non-monoaminergic pharmacotherapy for TRD, is ketamine (RS-ketamine) which is a racemic mixture of (S)-ketamine (esketamine) and (R)-ketamine (arketamine) isomers [243, 244]. Ketamine’s more potent derivative, esketamine, was recently approved for use as an adjunctive nasal spray for patients with TRD by both the U.S. Food and Drug Administration (FDA) and the European Medicines Agency (EMA) [245]. Ketamine is a noncompetitive NMDA receptor antagonist that induces a rapid and sustained relief of depressive symptoms [246], including the treatment-resistant subtype [141, 247]. Ketamine has a 35 – 60% response rate [245, 248, 249], and its antidepressant effects are rapidly realized (within 4 h) after a single dose, with a maximum effect attained between 24 to 72 h, and lasting for more than a week [32, 243, 250]. Unlike other antidepressants that require persistent administration, ketamine treatment typically starts with two doses per week, followed by a gradual decrease, which can result in the maintenance of approximately 40% of patients on a monthly or less frequent dose [250]. However, due to differential patient sensitivity to ketamine and variation in the rate of metabolism, the optimal prescription dosage is still unknown [250], but typically TRD patients are treated with 0.2—0.5 mg/kg, and a higher dose of 0.7—1 mg/kg is recommended for nonresponsive patients [251].

It is believed that the antidepressant effect of ketamine is mainly induced through antagonizing NMDARs on GABAergic interneurons [242, 252], which results in disinhibition of glutamatergic neurons and subsequent increase in extracellular glutamate levels [242]. The increase in glutamatergic transmission induces an increase in BDNF levels, which enhances synaptic activity and structural connectivity in the PFC [246, 253], and results in an antidepressant effect [250]. However, despite the highly prevalent belief that NMDAR antagonism is Ketamine’s mechanism of action, evidence shows that other NMDAR antagonists are not effective in relieving depression [243, 254, 255]. This suggests that NMDAR antagonism is not the only mechanism of action, and some studies have found that antagonism of AMPARs suppresses, while their activation significantly enhances the antidepressant effect of ketamine [256–258]. This suggests that the underlying mechanisms of action of ketamine involve activation and/or upregulation of AMPARs. Indeed, activation of AMPARs is associated with increased BDNF expression, which binds to TrkB receptors, and results in neurotrophin synthesis and subsequent increase in cortical connectivity and functioning [243, 250]. AMPAR is also associated with increased neurotransmitter levels and pro-inflammatory cytokines reduction [257, 259]. Thus the underlying mechanisms of action of ketamine in TRD, include NMDAR antagonism, AMPAR activation, increased BDNF synthesis, suppression of neuroinflammation, and possibly other still unknown biomarkers.

A recent ketamine-related discovery and of particular interest regarding the residual motivational symptoms that characterize TRD is that ketamine-induced increase in glutamate levels [249] and activation of µ-opioid receptors result in increased dopaminergic transmission [257, 260]. Indeed, a systematic review and meta-analysis confirmed that acute administration of ketamine in rodents induces a significant increase in DA levels in the cortex, striatum, and nucleus accumbens, and a 62–180% increase in the activity of dopaminergic neurons [261]. Ketamine rapidly increases DA levels in the medial prefrontal cortex, and antagonizing D_1_Rs in this region prevents the ketamine-induced rapid antidepressant effect [262, 263]. Thus, the finding that ketamine induces DA release [261, 264], and a rapid therapeutic action in TRD [265] with relief of the motor and motivational symptoms [36, 91, 99, 101, 102], further supports the assertion that the dopaminergic system is central in the etiology of TRD and may be involved in the fast therapeutic action of antidepressants that target other neurotransmitter systems.

However, despite its demonstrated effectiveness, recent meta-regression evidence shows that the efficacy of ketamine is not consistent across TRD populations [249, 252] and it is minimal in some patients [252]. There are also still concerns about the safety and tolerability of ketamine [245], which is for instance, associated with serious hallucinogenic [266], and dissociative side effects [250], and a high risk of suicide upon discontinuation of use [267, 268]. Another drawback is the safety profile of ketamine and its high potential for abuse and misuse [260], which is consistent with the conceivable involvement of DA, which is the main neurotransmitter in the regulation of mood, motivation, and reward-related behavior. Ketamine’s high potential for abuse, its associated dissociative symptoms, and other adverse side effects have prompted some countries to only permit the prescription of ketamine under a Risk Evaluation and Mitigation Strategy (REMS) that emphasizes a restricted distribution system [269]. Thus, more research is needed to assess the therapeutic efficacy, safety, and tolerability of ketamine when used independently or in combination with other antidepressant drugs and/or psychotherapy.

### Psychedelic-assisted therapy

The emerging therapies for TRD include psychedelics like psilocybin, which the FDA recently recognized as a breakthrough therapy for depression [270]. Naturally occurring in psilocybin mushrooms and structurally similar to endogenous 5-HT, psilocybin is a non-selective 5-HT_2A_R [271] and 5-HT_1A_R agonist [272]. Evidence shows that 5-HT_2A_R agonists increase the release of DA and NE in a rat frontal cortex [273]. Through activation of 5-HT_1A_Rs and 5-HT_2A_Rs in humans, psilocybin induces an increase in DA levels, psychotic-like symptoms [272], and an antidepressant effect [274]. In augmentation with psychological support, psilocybin induces rapid and long-lasting antidepressant effects, which can last for 6 [275] to 12 months after the last treatment session [276]. A recent study found that 50% of TRD patients who received two doses of psilocybin, taken a week apart, had a rapid relief of depressive symptoms that lasted for 3 months [277]. The finding that through activation of 5-HT receptors, psilocybin increases DA release, and relieves TRD symptoms, further underscores the central role of the dopaminergic system in the pathogenesis and treatment of depression.

### Anti-inflammatory therapies

Among the promising anti-inflammatory therapies for TRD, are cytokine inhibitors or monoclonal antibodies targeting specific pro-inflammatory cytokines. A recent meta-analysis of RCTs with 2370 participants found that anti-cytokine therapies such as infliximab, adalimumab, etanercept, and tocilizumab, induce a significant antidepressant effect [278]. These anti-cytokine therapies and other anti-inflammatory agents including NSAIDs, glucocorticoids, statins, and minocycline [279] are promising antidepressants, and seem to be particularly effective for a subgroup of depression patients with high levels of pro-inflammatory mediators. A recent meta-analysis revealed that celecoxib and minocycline, are currently the most effective among the promising anti-inflammatory treatments for depression [280, 281]. Celecoxib, is a cyclooxygenase-2 (COX-2) inhibitor, that suppresses pro-inflammatory cytokine release [203], while minocycline, a potentially effective antidepressant for TRD, is a tetracycline antibiotic with anti-inflammatory, antioxidant, and neuroprotective properties [282]. A recent meta-analysis of 28 RCTs with 3394 participants indicated that used independently, and in augmentation, anti-inflammatory agents including NSAIDs, N-acetylcysteines (NACs), corticosteroids, monoclonal antibodies, minocycline, statins, pioglitazone, and omega-3 fatty acids, induce significant antidepressant effects compared to controls [283]. These anti-inflammatory therapies provide promise for targeted treatment strategies for TRD, which may, for example, involve the identification of elevated cytokine subtypes, and the use of specific anti-cytokine therapies.

### HPA axis targeting therapies

As earlier stated, TRD is associated with HPA axis hyperactivation, which induces an over-secretion of cortisol, which desensitizes GRs, and results in impairment of the negative feedback loop, and subsequent treatment-resistant depression symptoms. This has resulted in an increasing interest in the HPA axis as a potential novel therapeutic target in TRD [203]. Indeed, a recent meta-analysis of 16 RCTs and 7 open-label studies with a total of 2972 participants, found mifepristone, a GR antagonist, to be an effective antidepressant [284]. Evidence also shows that metyrapone, a cortisol synthesis inhibitor, induces a significant reduction in depressive symptoms [284]. An RCT with a 3-week augmentation of metyrapone with SSRIs revealed a 50% antidepressant effect 5 weeks after the initiation of treatment [285]. These results suggest that inhibition of the HPA axis via regulation of cortisol release or activity could be an effective TRD treatment strategy.

### Dopaminergic therapies

The most prominent features of TRD including anhedonia and reduced motivation [36, 91, 99, 101, 102], are believed to largely be a result of dysfunction in the dopaminergic system, which when targeted tends to induce fast and long-lasting antidepressant effects. For example, DA agonists inhibit the production of pro-inflammatory cytokines through interaction with DA receptors on immune cells [286], and administration of DA receptor agonists [287] relieves the motivational symptoms that characterize TRD. An RCT assessing the efficacy of pramipexole, a DA receptor agonist, among TRD patients who were unresponsive to SSRIs, found that when used in augmentation with SSRIs it induced a 40% response, and 33% remission, while in another study it was associated with a 71% response, and 59% remission rate [288]. Furthermore, presynaptic D_2_/D_3_ autoreceptor antagonists, such as amisulpride, have a rapid onset of antidepressant effect, and can effectively address high-grade treatment resistance when used as augmentation agents [92]. Indeed in this review, dopamine appears to be a common or convergent mechanism in antidepressant treatment response, and future research on pharmacological interventions for TRD could focus on dopamine as a central variable.

### Limitations

This review integrates evidence from multiple study types, including primary studies, reviews, meta-analyses, and RCTs. Although the integration of evidence from a variety of studies provides a holistic perspective and potential for enhanced generalizability of the findings, their heterogeneity might be a major limitation when comparing the findings. Furthermore, the reviewed publications were identified based on their relevance to the topic, and not through systematic methods that would minimize the potential literature search bias, and increase the replicability of the findings. Another limitation is that this review only focused on studies published in English, and thus excluded publications in other languages. Furthermore, a number of studies on the recently discovered antidepressant therapies were limited to the documentation of observed response and remission rates, without enough evidence on the yet-to-be-discovered mechanisms of action and long-term therapeutic effects. This implies that some of the evidence from these studies might have been based on premature analyses of therapeutic outcomes. Future endeavors should utilize systematic reviews with replicable methods for searching and synthesizing evidence from specific study types. The systematic reviews could yield more enlightening and generalizable evidence about the underlying pathophysiology of depression and treatment modalities.

### Conclusions and future directions

Despite extensive research efforts over the past 60 years, the exact underlying mechanisms in the pathogenesis of depression and antidepressant treatment response are still unknown. This review shows that depression is a heterogeneous disorder, caused by multiple interactive molecular mechanisms, which seem to differ depending on various factors such as genetic vulnerability, and stressful life events. This variability in the interactive underlying mechanisms not only accounts for the different subtypes of depression but also contributes to the limited success in identifying precise therapeutic targets. This has led to continued reliance on trial-and-error treatment strategies and a lack of therapeutic efficacy of first-line conventional antidepressants. The ineffectiveness of antidepressants is observed in about 66% of patients, and might largely be a result of the tendency to target specific mechanisms, such as 5-HT deficiency, without consideration of the other interactive etiological mechanisms. Indeed, whereas SSRIs are the most commonly used antidepressants, they are not effective for many patients, and their mechanism of action, which is increased 5-HT levels, can interact with the DA and NE systems and induce or worsen certain depression symptoms. In particular, exclusive SSRI treatment increases 5-HT-mediated tonic inhibition of DA and NE neurotransmission, with subsequent inducement or worsening of anhedonia, motivational deficits, and other DA and NE deficiency-associated depressive symptoms. This phenomenon explains the observation that exclusive SSRI treatment tends to be associated with treatment-resistant or residual psychomotor, anhedonic, motivational, and other reward-related symptoms. It also underscores the need for considering the interactive neurotransmitter systems and other underlying mechanisms, which can exacerbate the adverse side effects of prescribed antidepressants, and complicate treatment.

This review shows that the etiology of TRD involves complex interactions among stress hormones, pro-inflammatory cytokines, neurotransmitter systems, genetics, and other factors. Target-specific therapy may not induce significant therapeutic effects, except where the therapeutic target is a convergent mechanism in this intricate interaction among etiological factors. Thus the development of efficacious antidepressant therapy requires extensive research guided by interactive mechanisms hypotheses, with emphasis on the heterogeneity of depression and the multiple underlying interactive processes. Such studies should assess the feasibility of utilizing multimodal or convergent mechanisms-specific antidepressant therapy. The advantage of evidence-based multimodal antidepressants is that they can target several etiological mechanisms, with the potential for optimal relief of depression while minimizing the reliance on the currently utilized trial-and-error switching, augmentation, and combination of antidepressants. However, targeting several interactive mechanisms has the potential for increased risk of adverse side effects, and extensive research is needed to identify the most feasible multimodal pharmacotherapy.

The potential side effects of multimodal antidepressants could be minimized through augmentation with psychotherapy or other non-pharmacological therapeutic modalities with minimal or no interaction with the prescribed medication. Since somatic therapies such as ECT, VNS, and TMS can induce alterations in neurotransmitter levels they could potentially increase the risk of side effects if used in argumentation with multimodal antidepressant medication. The need for these side effects-prone multimodal antidepressants could be minimized through the use of therapeutic agents that precisely target the convergent mechanisms. This review identifies DA deficiency, as a possible convergent mechanism in the etiology of TRD and antidepressant treatment resistance. For example, DA deficiency increases the release of pro-inflammatory cytokines, which induce various processes linked to the pathophysiology of depression, including the depletion of 5-HT, DA, NE, and GABA, increased glutamate levels, and decreased BDNF-TrkB signaling. Indeed, the most prominent features of TRD, including anhedonia, and motivational deficits, are associated with DA deficiency. Functional deficiency in the DA system is associated with reduced responsiveness to conventional antidepressants, including SSRIs, TCAs, and SNRIs, and the therapeutic effects induced by their prolonged use are reversed by low doses of DAR antagonists. On the other hand, direct targeting of the DA system induces rapid antidepressant therapeutic effects, and augmentation of conventional antidepressants with non-ergot D_3_R agonists like pramipexole, or D_2_/D_3_ autoreceptor antagonists like amisulpride, seems to be an effective treatment for TRD. Thus targeting the DA system as a convergent mechanism in the etiology of TRD could minimize the need for multimodal antidepressants, and result in better therapeutic outcomes. Future research could focus on the feasibility of using dopaminergic agents as either standard-alone therapy or in combination with other antidepressant therapies with minimal potential for adverse side effects.

This review indicates that the therapeutic effects of antidepressant drugs require adaptive changes such as the desensitization of D_2_/D_3_ autoreceptors, and 5-HT_2C_Rs, on the DA and NE neurons, and 5-HT_2A_Rs on GABAergic interneurons, which might account for the delayed onset of the therapeutic effects of conventional antidepressants. The duration taken for these adaptive changes to take place may greatly differ depending on several factors, such as the drug type and genetics, and it ranges from several days to months. Consequently, there is no consensus on the duration of treatment failure, to be used as a criterion for identifying TRD patients. Thus clinicians may not be in a position to quickly identify treatment-resistant patients, and make timely decisions regarding the best alternative treatment options. Most experts consider a duration of 4 weeks, but this review shows that about 20% of the patients who do not respond to treatment during the first 4 weeks, can respond after 5 to 8 weeks, while about 10% can respond after 9 to 12 weeks. Thus there is great variability in the duration taken for antidepressants to induce therapeutic effects and for some patients, it might require several months. This suggests that some individuals may be diagnosed as TRD patients and given alternative prescriptions, yet they would have benefited from first-line conventional antidepressants, after a prolonged duration of use. However, it might seem very difficult for clinicians to maintain their patients on the same seemingly ineffective prescription for several weeks or months. Thus future research could explore the prospects of developing therapeutic agents that have the potential for accelerating the adaptive changes such as the downregulation of D_2_/D_3_ autoreceptors and 5-HT_2C_Rs, and upregulation of BDNF synthesis, required for antidepressants to exert their therapeutic effects. This might not only result in rapid therapeutic effects, but could also minimize the reliance on the current trial-and-error switching, augmentation, or combination of drugs.

In summary, the lack of therapeutic efficacy of the current antidepressants could in part be attributed to the common assumption that certain specific mechanisms such as 5-HT or NE deficiency, are the underlying causes of depression, and the tendency to rely on this assumption as a basis for antidepressant drug development. Contrary to this assumption, depression is a heterogeneous disorder with multiple underlying etiological mechanisms, whose interaction results in variability in its manifestation and treatment response. Thus unless proven to be convergent biomarkers in the interaction, focusing on one or two etiological mechanisms as the therapeutic targets may not be sufficient, and could account for the current limited success in discovering antidepressants with reliable therapeutic efficacy. Effective management of depression might require targeting the interactive mechanisms or their convergent processes, through subtype-specific and/or personalized therapeutic modalities. The therapy could, for example, involve a combination of evidence-based multi-target pharmacotherapies, augmented with psychotherapy and/or other non-pharmacological approaches, with a potential for minimal adverse side effects. Future research could explore the prospects for developing multimodal, or convergent mechanisms-targeting antidepressants, as well as agents that can accelerate the adaptive processes required to achieve antidepressant therapeutic effects.

## Data Availability

Not applicable. All data was obtained from published studies and it is included in the manuscript.
